# Supplementary material for: Diversity of bioprotective microbial organisms in Upper Region of Assam and its efficacy against Meloidogyne graminicola
Source: PeerJ. 2023 Jul 28;11:e15779. doi: 10.7717/peerj.15779 (PMC10389073; doi:10.7717/peerj.15779)
Supplement: Supplemental Information 3 [file peerj-11-15779-s003.docx]

**Supplementary Figures**

| **Bacterial Strain** | **Gram Staining** | **KOH test** | **Citrate test** | **Catalase test** |
| --- | --- | --- | --- | --- |
| **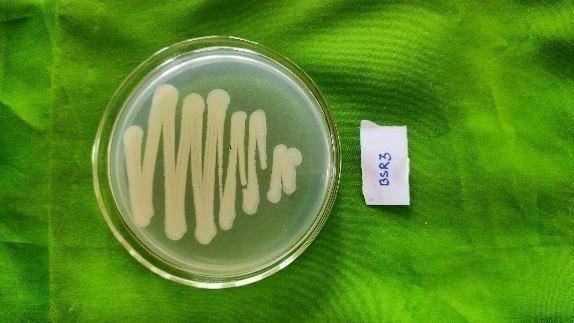** | **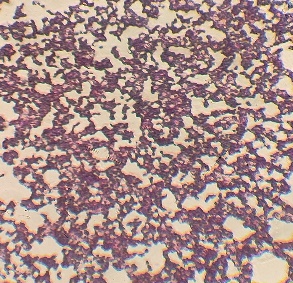** | **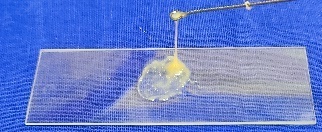** | **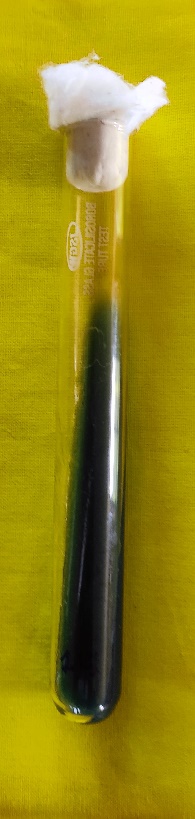** | **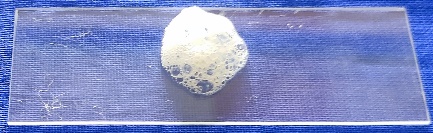** |
| **BTS4** | **+ve** | **-ve** | **+ve** | **+ve** |
| **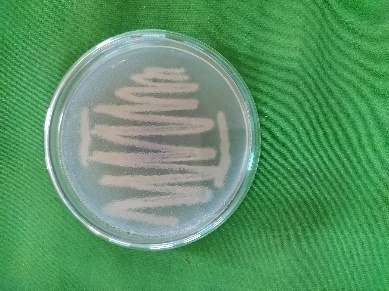** | **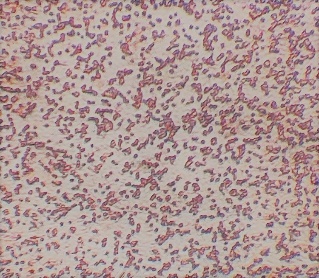** | **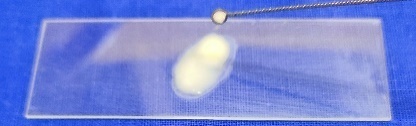** | **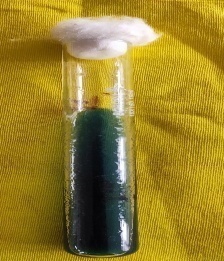** | **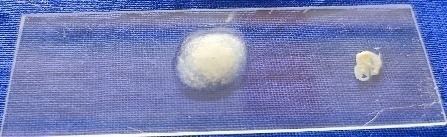** |
| **BTS5** | **-ve** | **+ve** | **-ve** | **+ve** |
| **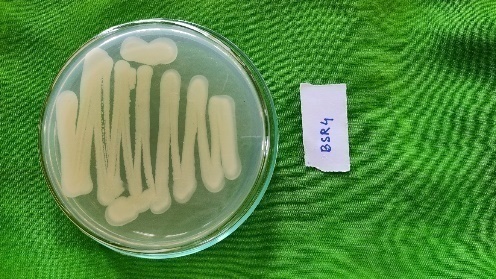** | **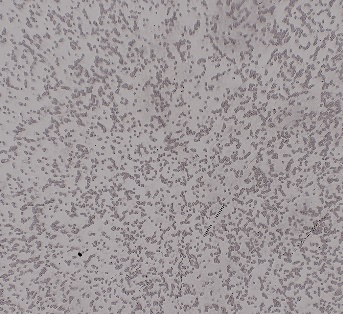** | **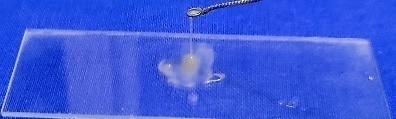** | **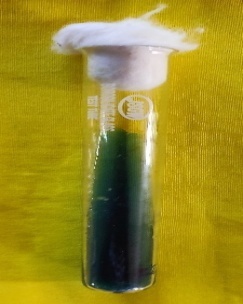** | **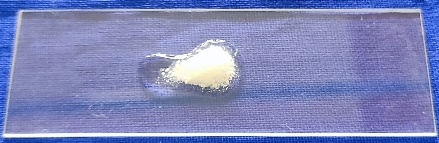** |
| **BSH8** | **+ve** | **-ve** | **+ve** | **+ve** |
| **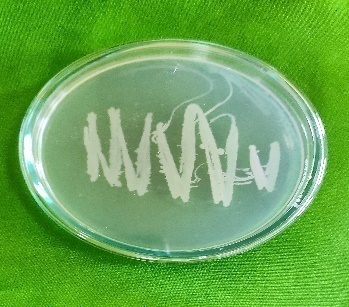** | **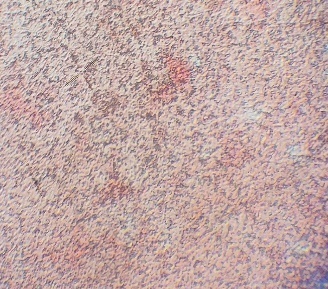** | **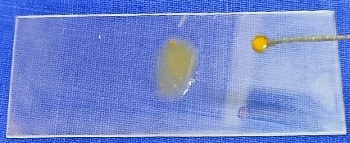** | **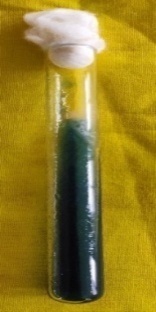** | **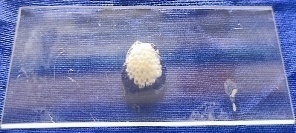** |
| **BJA15** | **-ve** | **+ve** | **-ve** | **+ve** |

**Supplementary Fig. 1: Documentation of promising bacterial strains from crop rhizosphere and their biochemical characterization.**


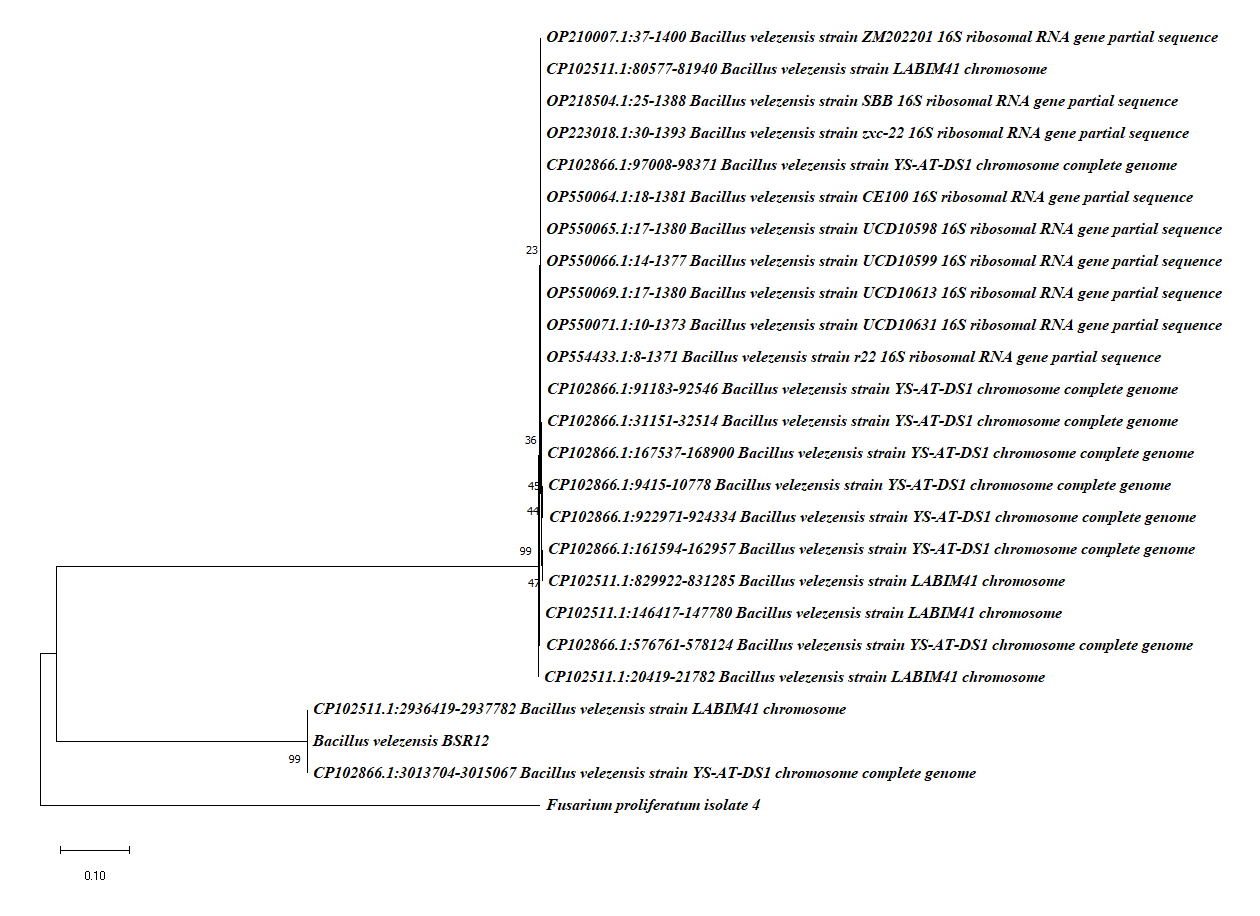


**Supplementary Fig 2. Phylogenetic tree of *Bacillus velezensis* with out-group and boot strap values.**

**
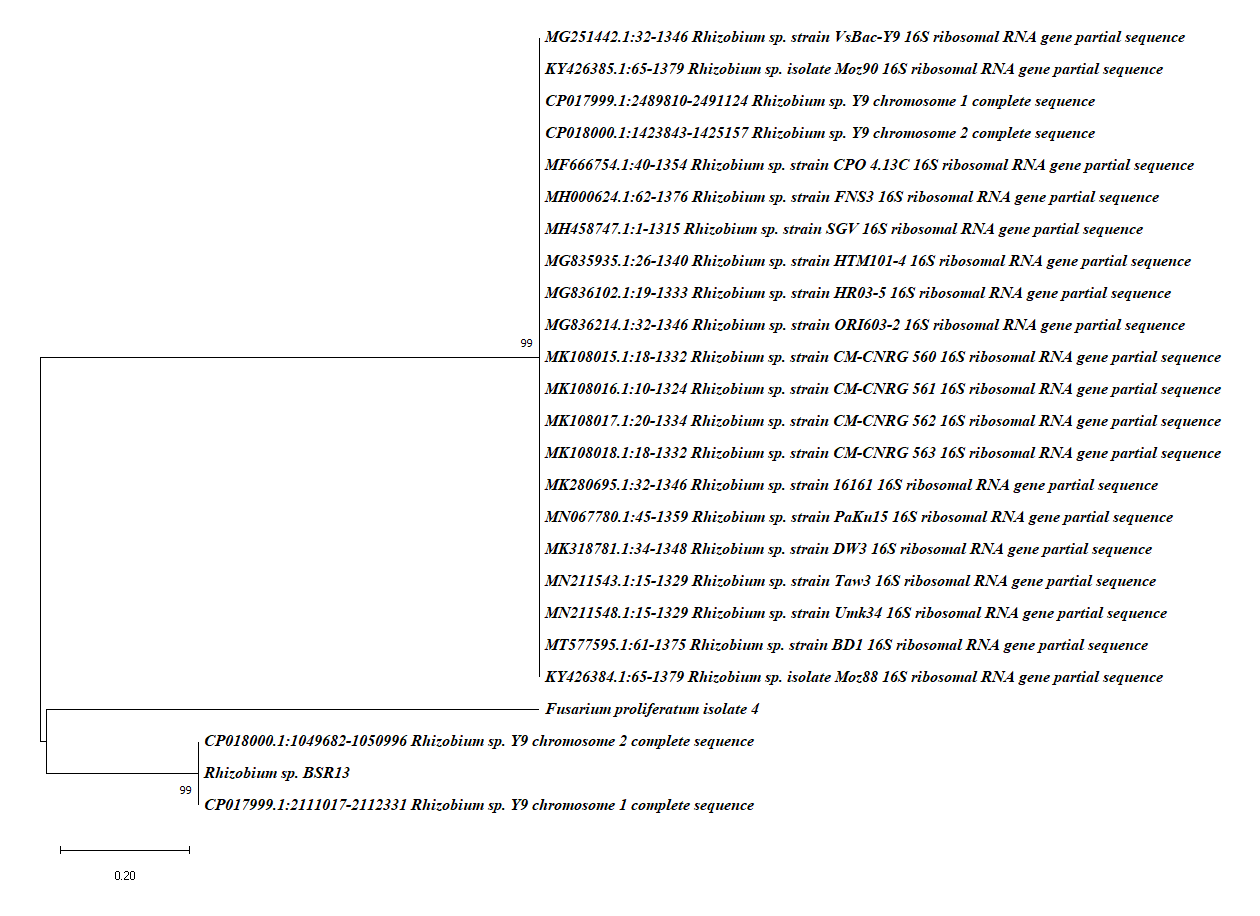
**

**Supplementary Fig 3. Phylogenetic tree of *Rhizobium sp.*  with out-group and boot strap values.**


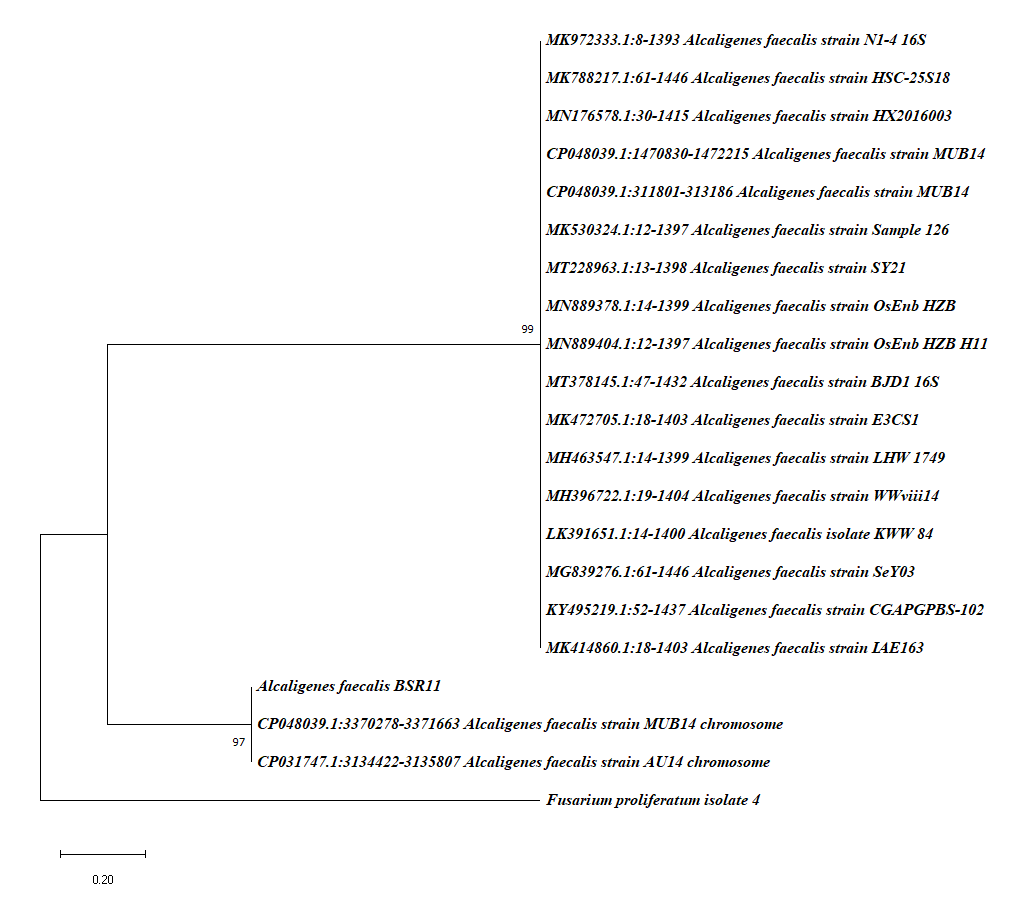


**Supplementary Fig 4: Phylogenetic tree of *Alcaligenes faecalis* with out-group and boot strap values.**

**Supplementary Fig 5: Phylogenetic tree of *Bacillus subtilis* with out-group and boot strap values.**

**
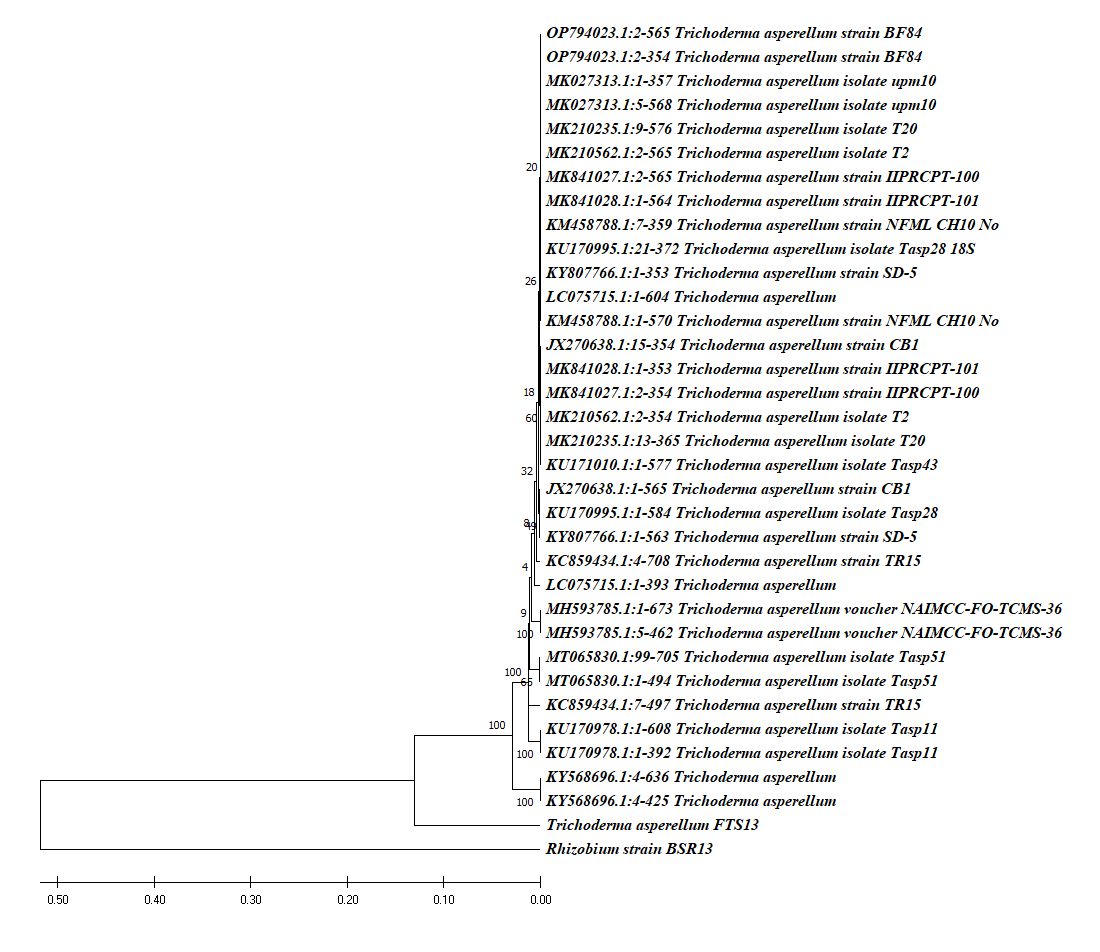
**

**Supplementary Fig 6: Phylogenetic tree of *Trichoderma asperellum* with out-group and boot strap values.**


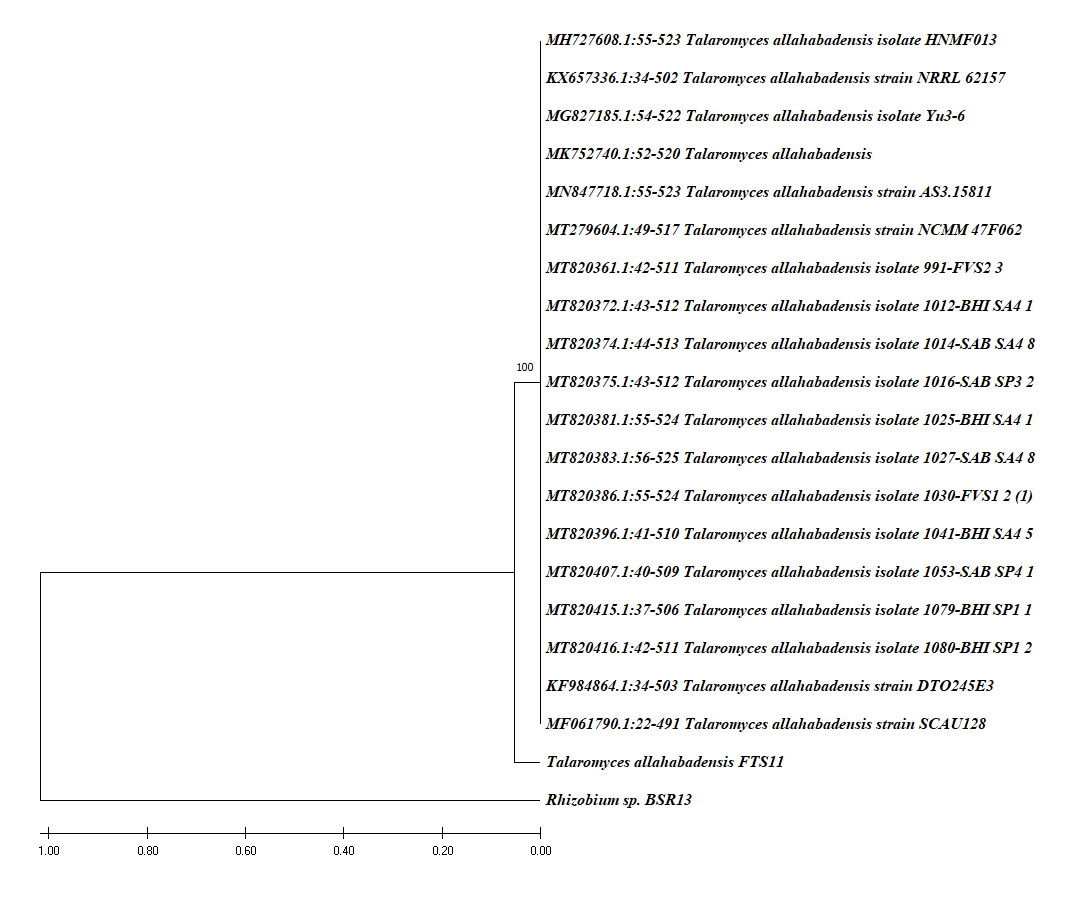


**Supplementary Fig 7: Phylogenetic tree of *Talaromyces allahabadensis* with out-group and boot strap values.**
