# Supplementary material for: Diversity of bioprotective microbial organisms in Upper Region of Assam and its efficacy against Meloidogyne graminicola
Source: PeerJ. 2023 Jul 28;11:e15779. doi: 10.7717/peerj.15779 (PMC10389073; doi:10.7717/peerj.15779)
Supplement: Supplemental Information 5 [file peerj-11-15779-s005.docx]

| **Sl.no** | **Code** | **Accession numbers** | **Name of Organism** | **Submitted Source** |
| --- | --- | --- | --- | --- |
| 1. | BTS4 | OQ216889 | *Bacillus velezensis* | NCBI |
| 2. | BTS5 | OQ216890 | *Alcaligenes faecalis* | NCBI |
| 3. | BSH8 | OQ216891 | *Bacillus subtilis* | NCBI |
| 4. | BJA15 | OQ216892 | *Rhizobum pusense* | NCBI |
| 5. | FSH5 | OQ244365 | *Talaromyces allahabadensis* | NCBI |
| 6. | FJB11 | OQ244366 | *Trichoderma asperellum* | NCBI |

TableS1: Accession numbers of the identified potential organisms
